# Expression of a Peroral Infection Factor Determines Pathogenicity and Population Structure in an Insect Virus

**DOI:** 10.1371/journal.pone.0078834

**Published:** 2013-11-05

**Authors:** Oihane Simón, Trevor Williams, Martine Cerutti, Primitivo Caballero, Miguel López-Ferber

**Affiliations:** 1 Instituto de Agrobiotecnología, Consejo Superior de Investigaciones Científicas-Gobierno de Navarra, Mutilva Baja, Navarra, Spain; 2 Instituto de Ecología AC, Xalapa, Veracruz, Mexico; 3 Laboratoire Baculovirus et Thérapie, Centre national de la recherche scientifique, Saint Christol-Les-Alés, France; 4 Departamento de Producción Agraria, Universidad Pública de Navarra, Pamplona, Spain; 5 Laboratoire de Génie de l'Environnement Industriel, Ecole des mines d'Alès, Alès, France; Natural Resources Canada, Canada

## Abstract

A Nicaraguan isolate of *Spodoptera frugiperda* multiple nucleopolyhedrovirus is being studied as a possible biological insecticide. This virus exists as a mixture of complete and deletion genotypes; the latter depend on the former for the production of an essential *per os* transmission factor (*pif1*) in coinfected cells. We hypothesized that the virus population was structured to account for the prevalence of *pif1* defector genotypes, so that increasing the abundance of *pif1* produced by a cooperator genotype in infected cells would favor an increased prevalence of the defector genotype. We tested this hypothesis using recombinant viruses with *pif1* expression reprogrammed at its native locus using two exogenous promoters (*egt*, *p10*) in the *pif2*/*pif1* intergenic region. Reprogrammed viruses killed their hosts markedly faster than the wild-type and rescue viruses, possibly due to an earlier onset of systemic infection. Group success (transmission) depended on expression of *pif1*, but overexpression was prejudicial to group-specific transmissibility, both in terms of reduced pathogenicity and reduced production of virus progeny from each infected insect. The presence of *pif1*-overproducing genotypes in the population was predicted to favor a shift in the prevalence of defector genotypes lacking *pif1*-expressing capabilities, to compensate for the modification in *pif1* availability at the population level. As a result, defectors increased the overall pathogenicity of the virus population by diluting *pif1* produced by overexpressing genotypes. These results offer a new and unexpected perspective on cooperative behavior between viral genomes in response to the abundance of an essential public good that is detrimental in excess.

## Introduction

Alphabaculoviruses (lepidopteran infecting nucleopolyhedroviruses) are insect pathogens, some of which form the active ingredient in a number of bioinsecticidal products [1], [2]. These viruses produce two types of virions: budded virions (BVs) for cell-to-cell transmission in infected insects and occlusion derived virions (ODVs) that are occluded within occlusion bodies (OBs) for insect-to-insect transmission. A number of *per os* infection factors (PIFs) are necessary for primary virus infection that involves binding to microvilli receptors and fusion between the ODV membrane and the microvilli of midgut cells [3]. This is a multistep process [4] that seems to involve a highly stable multimolecular complex of PIF factors [5], [6].

PIF1 was first identified at a very low level in ODV membranes [7]. The reason for the very low expression of *pif1* [8] is uncertain, but might be related to some unique property of the protein. The level of expression of *pif1* compared to that of other *pif* genes is unknown, although the amount of *pif1* mRNA transcript was estimated to be 300 times lower than that of *polh* mRNA. In a *Spodoptera frugiperda* multiple nucleopolyhedrovirus population defective genotypes, SfNIC-C and –D, are not infectious *per os* due to a 16.4 kb deletion that includes the *pif1* and *pif2* genes. These defective genotypes survive by complementation with *pif1*/*pif2*-containing genotypes in cells infected by multiple genotypes [9], [10].

Occlusion bodies (OBs) of SfNIC-B, the dominant genotype in the population and the genotype with the largest genome [9]–[11], are less pathogenic than OBs of the wild-type mixture, in terms of concentration-mortality metrics. However when ODVs of complete and defective genotypes were mixed in near natural proportions (75% SfNIC-B:25% SfNIC-C) and co-occluded into OBs, the pathogenicity of the mixed genotype OBs was restored to that of the wild-type population [10], [12]. Moreover, when subjected to serial passage in insects, mixed genotype OBs, comprising non-natural proportions of complete and defective genotypes, rapidly converged to a common stable proportion that reflected the natural proportion of each type of genotype, suggesting that the wild-type population is genetically structured to increase the likelihood of transmission [9], [10], [12]–[14].

Near identical results were observed in experiments with mixtures of SfNIC-B and recombinant viruses based on SfNIC-B in which *pif1* and *pif2* had been deleted, indicating that the absence of *pif1* and *pif2* in a fraction of the population is both necessary and sufficient to explain the observed pathogenicity phenotype of mixed genotype OBs [15]. It seems that *pif1*/*pif2* expression is regulated not only at transcriptional level but also at population level, accounting for a higher prevalence of defective genotypes, to maximize the transmissibility of the OBs. Accordingly, Clavijo et al. [15] predicted that enhancing *pif1* expression would have two different effects. First, a reduction in the potency of OBs of the reprogrammed genotype due to an increase in the amount of PIF1 in ODVs that could adversely influence ODV entry into midgut cells. Second, a shift in the frequencies of *pif1* reprogrammed and deletion genotypes in mixed infections would be required to restore OB potency to that of the wild-type population.

In the present study we explored the consequences of manipulating the expression of *pif1* that represents a public good in cells infected by multiple genotypes. We examined the hypothesis that expression of this gene alters the pathogenicity of OBs and thereby determines the frequencies of cooperator and defector genotypes in the virus population. To test this, the weakly transcribed *pif1* gene [7], [8] was reprogrammed under the control of an early promoter (*egt* promoter) [16], [17], or a strong late promoter (*p10* promoter) [18], [19] originating from a closely-related nucleopolyhedrovirus. The pathogenicity of OBs produced in insects infected by mixtures of reprogrammed and defector genotypes was then analyzed and shown to follow the predicted response.

## Materials and Methods

### Insects, cells and viruses

Larvae from a laboratory colony of *S. frugiperda* were maintained on a wheatgerm-based semisynthetic diet [20] at 25°C. Sf9 cells were cultured at 28°C in TC100 medium supplemented with 10% fetal calf serum (FCS), penicillin (1 U/ml) and streptomycin (1 µg/ml). Occlusion bodies (OBs) of a Nicaraguan isolate (SfNIC) of SfMNPV were amplified in *S. frugiperda* fourth instars. The complete genotype, SfNIC-B, was obtained from plaque purified material [9] and was used to construct the bacmid SfNIC-BΔ*pifs*, a virus with a 2.8 kb deletion encompassing the consecutive *pif1* and *pif2* genes [15].

### Construction of promoter-exchange donor vectors

**Figure 1:**
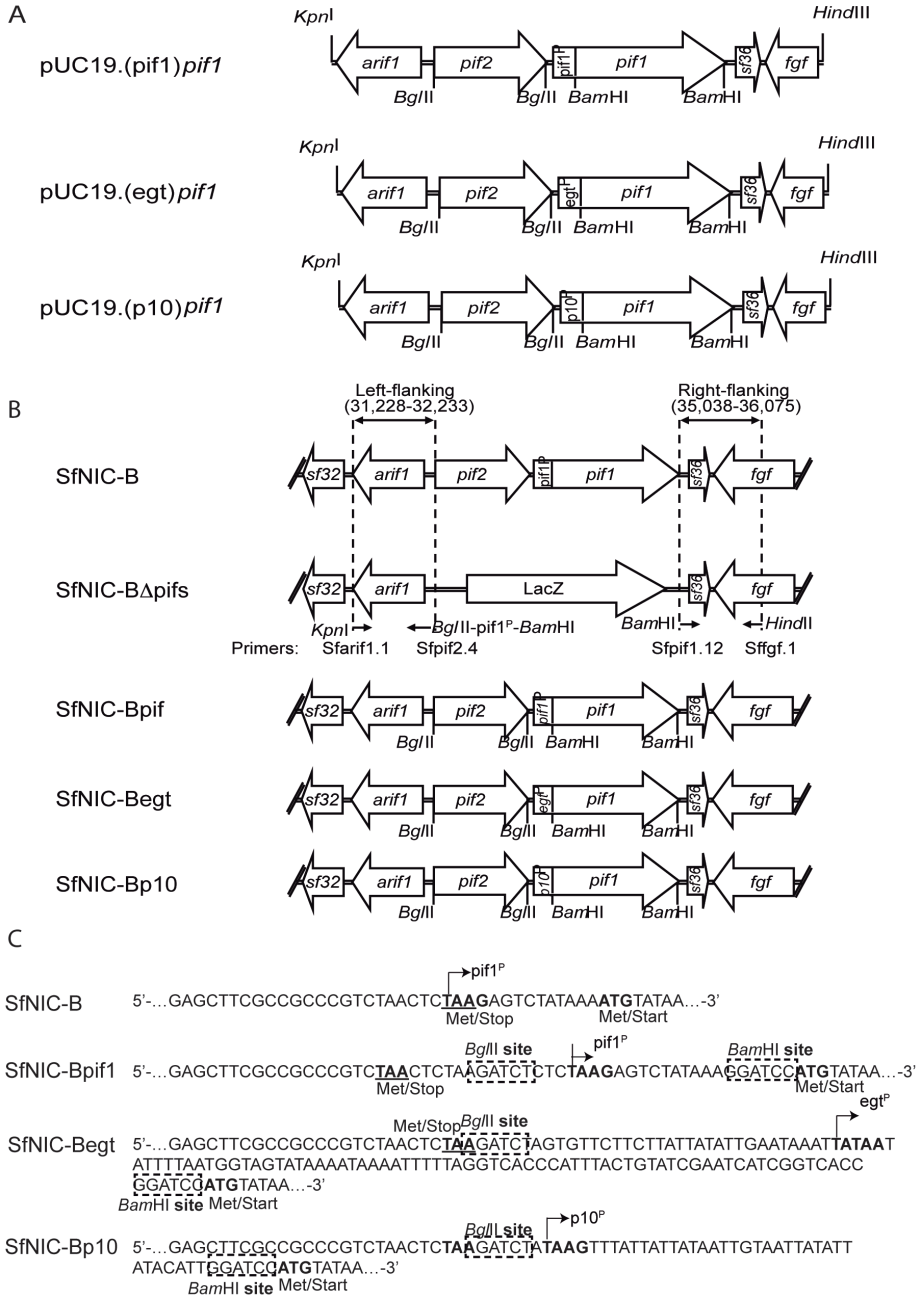
Promoter-exchange donor constructs and intergenic viral sequences. A) Donor plasmids used in this study. B) Viruses used in this study. The sequence of the complete genotype SfNIC-B is shown, where the right and left *pif-2*/*pif-1* flanking regions are specified. First, a SfNIC-BΔpifs recombinant virus was constructed in which *pif-2/pif-1* coding region was substituted by the LacZ operon. The primers used for the flanking regions amplifications are indicated below the figure. Recombinant viruses SfNIC-Bpif1 (rescue virus), SfNIC-Begt, in which the SeMNPV *egt* promoter would drive *pif-1* transcription, and SfNIC-Bp10, in which the SeMNPV *p10* promoter would drive *pif-1* transcription, were constructed by homologous recombination between SfNIC-BΔpifs and donor plasmids pUC19.(pif1)pif1, pUC19.(egt)pif1 and pUC19.(p10)pif1 respectively. C) *pif-2*/*pif-1* intergenic sequences from SfNIC-B, SfNIC-Bpif1, SfNIC-Begt and SfNIC-Bp10 viruses. The underlined portion in SfNIC-B containing the native *pif-1* promoter was replaced by donor sequences.

A pUC19-based transfer vector was constructed to insert the selectable *pif1* and *pif2* genomic region (nt 31,228 to 36,075) in the SfNIC-B genome [11] (accession number HM595733), that would subsequently be used to insert alternative *p10* or *egt* promoters from *Spodoptera exigua* NPV (SeMNPV), into the SfMNPV *pif2*-*pif1* intergenic region by homologous recombination (Fig. 1A). The primers used for the constructions are listed in Table S1. First, a plasmid was constructed that contains the left and right flanking regions of *pif2* and *pif1* from the SfMNPV genome. The left genomic-flanking region (1,006 bp; 31,228–32,233) of the donor cassette, amplified from SfNIC-B DNA using the Sfarif1.1/Sfpif2.4 primer sets, contained the full *arif1* ORF and partial downstream *sf32* ORF of unknown function. This genomic flanking region is located just upstream from the ATG start codon of *pif2*. The Sfpif2.4 primer sequence included a 30 bp (32,204-32,233) homologous region to the sequence upstream from the ATG of *pif2* and a *Bgl*II restriction site, the promoter region of *pif1* (33,431–33,447), and a *Bam*HI restriction site. The introduced *Bgl*II restriction site was used to clone the *pif2* ORF, whereas the *Bam*HI site was inserted to favor ligation to the right genomic-flanking region, and afterwards used to clone the *pif1* ORF. First, the *sf32*/*arif1* containing PCR fragment was cloned into the multiple cloning site of pUC19 using the PCR primer-introduced *Kpn*I and *Bam*HI restriction sites to create the plasmid pUC19.*sf32/arif1*. The right genomic-flanking fragment (1,038 bp; 35,038–36,075), amplified from SfNIC-B DNA using the Sfpif1.12/Sffgf.1 primer set, contained the complete *sf36* ORF of unknown function and the 3′ end of the *fgf* ORF. The *sf36*/*fgf*-containing amplicon was then cloned into the remaining MCS of pUC19.*sf32*/*arif1* using the PCR primer-introduced *Bam*HI and *Hin*dIII restriction sites. This plasmid, containing the right and left genomic flanking regions of the *pif2*/*pif1* genes, was designated as pUC19.*sf32/arif1-sf36/fgf*. The complete *pif2* gene (32,234–33,430) was amplified from SfNIC-B genome using the Sfpif2.5/Sfpif2.6 primer set. The *pif2*-containing PCR fragment was cloned into the pUC19.*sf32*/*arif1-sf36/fgf* plasmid using the primer-introduced *Bgl*II restriction site. The *pif1* gene (33,448–35,037) was also amplified from the SfNIC-B genome using the Sfpif1.13/Sfpif1.14 primer set, and cloned into pUC19.sf32/*arif1-pif2-sf36/fgf* utilizing the introduced *Bam*HI restriction site. The plasmid containing the right and left flanking regions and both *pif* genes, pUC19.*sf32*/*arif1-pif2-pif1-sf36/fgf* (designated pUC19.(pif1)*pif1* in Fig. 1A; the parentheses indicate the promoter, whereas the coding sequences are indicated in italics) was used to construct the donor plasmids for the cotransfection with the SfNIC-BΔ*pifs* virus [15].

The SeMNPV *egt* promoter (26,828–26,927) [20] was amplified by PCR from SeMNPV DNA using the SePregt.F-SePregt.R primer set (Table S1). The *egt* promoter PCR fragment containing primer-introduced *Bgl*II and *Bam*HI sites was cloned to the pUC19.*sf32/arif1-pif2-pif1-Sf36/fgf Bam*HI-digested plasmid, thus generating pUC19.*Sf32/arif1*-*pif2*-(egt)*pif1-Sf36/fgf* (designated pUC19.(egt)*pif1* in Fig. 1A), so that the *egt* promoter would drive *pif1* transcription. The sequence representing the SeMNPV *p10* promoter (123,702–123,739) [21] was designed as two complementary oligomers (SePrp10.F-SePrp10.R) (Table S1) that, when annealed, had compatible overhangs for ligation of the pUC19.*sf32*/*arif1-pif2-pif1-sf36/fgf Bgl*II-*Bam*HI digested plasmid, thus generating pUC19.*sf32/arif1-pif2*-(p10)*pif1-sf36/fgf* (designated pUC19.(p10)*pif1* in Fig. 1A), so that the *p10* promoter would drive *pif1* transcription. The integrity of all cloned sequences was verified by sequencing.

### Generation, isolation and screening of recombinant viruses

The DOTAP reagent and protocol (Roche, Basel, Switzerland) was used to cotransfect Sf9 cells with the LacZ^+^ SfNIC-BΔ*pifs* viral genomic and plasmid transfer pUC19.(pif1)*pif1*, pUC19.(egt)*pif1* and pUC19.(p10)*pif1* DNAs (Fig. 1A). OBs of three recombinant viruses were generated: (i) SfNIC-Bpif1, representing both a rescue virus and a positive control for the recombinant construction methodology, (ii) SfNIC-Begt, SfNIC-B genotype in which *pif1* was reprogrammed with the SeMNPV *egt* promoter and, (iii) SfNIC-Bp10, SfNIC-B genotype in which *pif1* was reprogrammed with the SeMNPV *p10* promoter (Fig. 1B). For this, cells were transfected with 1 µg of SfNIC-BΔ*pifs* genomic DNA and 5 µg of the corresponding plasmid transfer vectors (Fig. 1A). Viral plaques were screened by adding 30 ng/µl X-gal reagent to the TC100 medium. A total of 20 white plaques were picked from each transfection and each plaque was amplified in Sf9 cells. DNA was extracted from amplified plaques and the authenticity of the recombinant viruses was confirmed by sequencing of PCR amplicons spanning the *pif1* promoter region amplified using Sfpif1.7-Sfpif1.9 primers (Fig. 1C). OBs were produced by injecting 8 µl of each virus at 1×10^4^ pfu/ml in *S. frugiperda* fourth instars. The authenticity of OBs produced in insects was also confirmed by sequencing of the PCR products obtained following amplification using Sfpif1.7-Sfpif1.9 primers (Table S1).

### Temporal expression

Groups of 250 *S. frugiperda* second instars were inoculated with the 90% lethal concentration (LC_90_) of each of the following viruses SfNIC-B (1.65×10^6^ OBs/ml), SfNIC-Bpif1 (8.98×10^5^ OBs/ml), SfNIC-Begt (9.22×10^6^ OBs/ml), SfNIC-Bp10 (1.77×10^7^ OBs/ml) or mock-infected using the droplet feeding technique [22]. The experiment was performed three times. Total RNA was isolated from groups of 20 larvae at 0, 2, 4, 6, 8, 12, 24, 48 and 72 h post-infection (p.i.). The time zero h p.i. was defined as the moment that the larvae had ingested viral OBs. Total RNA was extracted from insect larvae using TRIzol isolation reagent (Invitrogen) according to manufacturer's protocol. The concentration and integrity of RNA samples were determined by measuring absorbance at 260 nm, and by agarose gel electrophoresis. RNA samples were stored at −80°C until required. The experiment was performed three times.

The temporal expression of *pif1* under the control of homologous or heterologous promoters, was determined by qRT-PCR. For this, 1 µg RNA was treated with DNase I (Promega) following manufacturer's instructions. cDNA was synthesized by using Improm-II™ Reverse Transcriptase (Promega), according to the manufacturer's protocol. The absence of contaminant DNA was verified by performing PCR without a prior reverse transcription step. Three sets of specific primers that annealed in *pif1* were designed based on the SfNIC-B genome sequence [11]. Non-template controls were analyzed for each set of primers designed in order to verify the absence of non-specific background signal. The qSfBpif1.F and qSfBpif1.R primer set (Table S1) was selected based on the presence of a single melting peak, an indicator of specific amplification. RNA isolated from mock-infected larvae, as well as the Milli-Q water used in all reactions, served as negative controls. All reactions were performed in triplicate.

A 1 µl volume of cDNA (1∶10 dilution) was used for qRT-PCR. All reactions were performed using SYBR Green fluorescence in an ABI PRISM 7900HT Sequence Detection System (Applied Biosystems). The reaction mixture (10 µl) contained 5 µl SYBR Premix Ex Taq (2×), 0.2 µl of ROX Reference Dye (50×), 0.1 µl of each SfMNPV primer (10 pmol/µl) (Table S1) and 1 µl of pooled cDNA. qPCR was performed under the following conditions: 95°C for 30 s, followed by 45 elongation cycles of 95°C for 5 s and 60°C for 30 s and finally a dissociation stage of 95°C for 15 s, 60°C for 15 s and 95°C for 15 s. Data acquisition and analysis were handled by Sequence Detector Version 2.2.2. software (Applied Biosystems).

SfNIC-B DNA was amplified in the *pif1* region by conventional PCR using the qSfBpif1.F and qSfBpif1.R primer set. The resulting product was electrophoresed in 1% agarose, excised and purified using a DNA purification kit (Macherey-Nagel, Duren, Germany). Purified DNA was cloned into the pGEM-T Easy Vector (Promega, Madison, WI, USA), and its identity was checked by PCR and restriction endonuclease analysis with *Pst*I. Volumes of 1 µl of plasmid DNA dilutions (10^−1^ to 10^−8^ ng/µl) were used as internal standards for each qPCR reaction. The number of target gene copies was calculated based on the DNA concentration and the molecular mass of the genome. Relative expression results at each time post-infection were subjected to analysis of variance (ANOVA) followed by Bonferroni means separation tests in SPSS ver. 17.0 (SPSS Inc.).

### Virus growth kinetics

To examine budded virus (BV) production, 3×10^5^ Sf9 cells were infected with 10 MOI of SfNIC-B, SfNIC-Bpif1, SfNIC-Begt and SfNIC-Bp10 BVs. Three supernatant samples were harvested from separate plates at 0, 2, 6, 12, 24, 48, 72, 96 and 120 h post-infection. Time zero was defined as the moment the virus inoculum was allowed to adsorb to the cells. The titers of supernatants were determined on Sf9 cells by end-point dilution [23]. Three independent infections were performed for each dilution. The experiment was performed three times. Results of BV production at different times post-infection were subjected to ANOVA followed by Bonferroni means separation tests; however for the samples taken at 2 h p.i. Kruskal-Wallis and Mann-Whitney tests were used as the data were not normally distributed. Critical α values were subjected to false discovery rate adjustment for multiple pairwise comparisons [24].

### Determination of phenotypic characteristics

The insecticidal properties of OBs produced after injection of larvae with BVs from SfNIC-B, SfNIC-Bpif1, SfNIC-Begt and SfNIC-Bp10 were determined by insect bioassay following the droplet feeding method [22]. Groups of *S. frugiperda* second instars were starved for 8-12 h at 25°C and were then allowed to drink from an aqueous suspension containing 10% (w/v) sucrose, 0.001% (w/v) Fluorella blue and one of the following five concentrations of OBs: 1.2×10^6^, 2.4×10^5^, 4.8×10^4^, 9.6×10^3^ and 1.9×10^3^ OBs/ml. This range of concentrations was previously determined to kill between 95 and 5% of the experimental insects [9]–[14]. Larvae that ingested the suspension within 10 min were transferred to individual wells of a 25-well tissue-culture dish with semisynthetic diet. Bioassays were performed three times using groups of 25 larvae per virus concentration and 25 mock-infected control larvae. Larvae were reared at 26°C and mortality was recorded every 8 h until insects had either died or pupated.

Virus induced mortality results were subjected to probit analysis using the Polo-Plus program [25]. OB pathogenicity was expressed as the 50% lethal concentration (LC_50_). Time mortality results of the four viruses were subjected to Weibull survival analysis in GLIM 4 [26]. As OBs of the different viruses differed significantly in potency, the OB concentrations used for the time mortality analysis were 1.65×10^6^ OBs/ml for SfNIC-B, 8.98×10^5^ OBs/ml for SfNIC-Bpif1, 9.22×10^6^ OBs/ml for SfNIC-Begt and 1.77×10^7^ OBs/ml for SfNIC-Bp10, that resulted in comparable mortalities of 85, 84, 86 and 84%, respectively. The experiment was performed four times.

OB production by each virus was determined *in vitro*. Infected Sf9 cells were harvested from the BV production experiment at different intervals post-infection, and were pelleted by low-speed centrifugation and washed once with 500 µl PBS. Cell pellets were resuspended in 25 µl TE and mixed with 25 µl of cell lysis buffer (50 mM Tris-HCl pH 8.0, 5% 2-mercaptoethanol, 0.4% w/v SDS, 10 mM EDTA). The resulting OB suspensions were quantified by direct counting in a bacterial counting chamber. OB counts from each suspension were performed three times. OB production results at 120 h p.i. were subjected to ANOVA followed by Bonferroni tests with false discovery rate adjustment for multiple pairwise comparisons [24].

OB production was also determined in insects. For this, *S. frugiperda* second instars that died from polyhedrosis disease in the time to death experiment were randomly selected from groups of 19–23 insects for each virus treatment in each repetition, representing a total of ∼80 larvae per virus treatment. Virus killed insects were individually stored at −20°C until used for OB counting. Each larva was thawed at room temperature, homogenized using a plastic pestle in a volume of 100 µl distilled water and serially diluted in distilled water. OB counts from each insect were performed in triplicate using a Neubauer hemocytometer. The results were normalized by logarithmic transformation and subjected to ANOVA and Bonferroni means separation.

### Physical characteristics of viral Obs

OBs of each virus were characterized for DNA content, nucleocapsid numbers per virion, and mean virion titer per OB. The DNA content of OBs was determined by qPCR. For this, OB suspensions containing 5×10^8^ OBs were mixed with 100 µl of 0.5 M Na_2_CO_3_, 50 µl of 10% SDS in a final volume of 500 µl and incubated for 10 min at 60°C. Undissolved OBs and other debris were removed by low-speed centrifugation (3,800 *x g*, 5 min). The supernant fraction containing released virions was treated with 25 µl of proteinase K (20 mg/ml) for 30 min at 50°C. Viral DNA was extracted twice with TE buffer (pH 8.0) saturated phenol and once with chloroform. Viral DNA was isolated by alcohol precipitation. The pellet was resupended in 100 µl of TE buffer for 10 min at 60°C. DNA samples in volumes of 1 µl were diluted 1∶100 and quantified by qPCR as previously described using the qSfBpif1.F/qSfBpif1.R primer set and standard curve. DNA was extracted from a total of nine samples and all reactions were measured in triplicate. The results were subjected to ANOVA and Bonferroni means separation.

To compare the distribution of numbers of nucleocapsids in virions of each virus, ODVs were harvested by adding 5×10^8^ OBs of each virus to an equal volume of 0.1 M Na_2_CO_3_. The resulting suspensions were layered onto a continuous 30–60% sucrose gradient and centrifuged at 76,800 *x g* for 1 h at 4°C in a Beckman Ti70 rotor. The banding patterns of each virus were visually inspected and photographed.

Mean numbers of ODV infectious units per OB were determined by end-point dilution as described previously [23]. Twenty four independent infections were performed for each dilution. The experiment was performed 12 times. Cells were examined daily for the presence of viral OBs in the nuclei for up to one week. TCID_50_ values were estimated by Spearman-Kärber method and were subsequently converted to infectious units per 5×10^8^ OBs for presentation in the figures.

### Production of OBs comprising co-occluded genotype mixtures

In order to determine the relationship between the proportions of recombinant and deletion viruses and OB potency, different co-occluded mixtures were created in which SfNIC-Begt:SfNIC-C and SfNIC-Bp10:SfNIC-C genotypes were co-enveloped into virions and subsequently co-occluded into OBs at the desired proportions following the methodology described previously [10], [12]–[14], in which ODVs released from OB mixtures were injected into *S. frugiperda* larvae. Previous studies demonstrated that co-envelopment of different genotypes in ODVs occurred following injection of mixtures of genotypes into larvae [27]. This technique was found to be effective for the production of mixed genotype OBs that contained each genotype in approximately the same to the proportions in which they had been injected. For this, OBs of each recombinant virus (SfNIC-Begt or SfNIC-Bp10) were diluted to a concentration of 5×10^8^ OBs/ml and were mixed with an identical concentration of deletion genotype SfNIC-C OBs in the following proportions: 90% recombinant:10% SfNIC-C, 75% recombinant:25% SfNIC-C, 50% recombinant:50% SfNIC-C, 25% recombinant:75% SfNIC-C, and 10% recombinant:90% SfNIC-C. ODVs were then released from OB mixtures by alkali disruption with a dissociation buffer (1 vol. OB suspension: 1 vol. 0.5M Na_2_CO_3_: 5 vol. H_2_O). Undissolved OBs were pelleted by low speed centrifugation at 2,700× *g* for 5 min. The ODV-containing supernatant was injected into groups of 50 *S. frugiperda* fourth instars (8 µl/larva). These larvae were individually maintained on semisynthetic diet until death. Extraction of OBs containing mixtures of co-occluded genotypes, OB purification and DNA extraction were then performed as previously described. DNA was extracted from nine independent samples of OBs.

qPCR reactions were performed to quantify SfNIC-Begt:SfNIC-C and SfNIC-Bp10:SfNIC-C ratios. For specific detection of SfNIC-Begt and SfNIC-Bp10 the qSfBpif1.F and qSfBpif1.R primers were used (Table S1). For detection of SfNIC-C, primers qSfCcath.F and qSfCsf36.F (Table S1) were designed around the 16.37 kb deletion that is characteristic of SfNIC-C genotype, located between nt 18,752 and 35,122 in the SfNIC-B genome [10], [11]. This primer set was selected based on the presence of a single melting peak. The SfNIC-C PCR product from a standard PCR reaction was cloned into pGEM-T Easy Vector as described above. Volumes of 1 µl of plasmid DNA containing the SfNIC-B and SfNIC-C PCR products were diluted (10^−1^–10^−8^ ng/µl), and used to construct standard curves. Non-template controls were also analyzed for each set of primers designed in order to verify the absence of non-specific background signal.

Prior to analysis, DNA samples from mixtures of SfNIC-Begt:SfNIC-C and SfNIC-Bp10:SfNIC-C OBs were used to calibrate the qPCR assay. DNA samples from SfNIC-Begt:SfNIC-C and SfNIC-Bp10:SfNIC-C OBs, and a range of mixtures (1∶10^3^-10^3^∶1) in 10-fold intervals were quantified by qPCR. In order to standardize the OB quantification, the amounts of DNA in the OBs of SfNIC-Begt, SfNIC-Bp10 and SfNIC-C were determined in a previous qPCR assay using the primers sets for SfNIC-Begt or SfNIC-Bp10 and SfNIC-C, described above. No significant differences were observed in the amounts of genomic DNA in samples of 5×10^8^ OBs between the different viruses (p>0.05). Triplicate samples of the calibration mixtures were also included with the SfNIC-Begt:SfNIC-C and SfNIC-Bp10:SfNIC-C co-occluded mixtures in the qPCR assay. All reactions were performed in triplicate.

## Results

### Genomic characterization of viruses

The identity of OBs produced in insects was confirmed by sequencing of the PCR products obtained following amplification using Sfpif1.7-Sfpif1.9 primers, which revealed that the genomic arrangement of the recombinant viruses SfNIC-B, SfNIC-Bpif1, SfNIC-Begt and SfNIC-Bp10 differed only at the *pif1*/*pif2* intergenic locus (Fig. 1C).

### Temporal transcription of *pif1* in reprogrammed viruses

Temporal regulation of *pif1* transcription was examined by quantitative RT-PCR (qRT-PCR) using total RNA isolated from infected *S. frugiperda* larvae at different times post-infection (Table 1). Control reactions, performed to ensure the absence of contaminant DNA, did not result in amplification. The efficiency of the qRT-PCR was 104% (r^2^ = 0.9916), which indicated that this technique generated accurate estimates of target nucleic acid copies [28]. Transcripts of *pif1* were first detected at a very low level at 12 h p.i. in *S. frugiperda* larvae infected with SfNIC-B or SfNIC-Bpif1 rescue virus, with estimated mean (±SD) copy numbers of 104±8.56 and 100±5.67 cDNA copies/µg total RNA, respectively. The abundance of *pif1* transcripts increased by approximately 25-fold at 72 h p.i., with copy numbers estimated at 2,838±183 or 2,559±175 cDNA copies/µg RNA in larvae infected with SfNIC-B or SfNIC-Bpif1 viruses, respectively. The transcription pattern of *pif1* in larvae infected with SfNIC-B or SfNIC-Bpif1 did not differ significantly between these viruses over time (ANOVA, Bonferroni test, p>0.05). In contrast, in larvae infected with SfNIC-Begt, *pif1* transcripts were detectable by 4 h p.i. (130±9.64 cDNA copies/µg RNA), increased by 6-fold (734±74 cDNA copies/µg RNA) and 2,700-fold (351,368±12,108 cDNA copies/µg RNA) between 6 and 72 h.p.i. Similarly, in larvae infected with SfNIC-Bp10, *pif1* transcription was detected at 24 h p.i. (2,583±154 cDNA copies/µg RNA), increasing 97-fold at 48 h.p.i. (250,850±18,936 cDNA copies/µg RNA) and by approximately 480-fold at 72 h p.i. (1,252,025±109,814 cDNA copies/µg RNA).

**Table 1:** Relative expression of *pif1* (cDNA copies/µg RNA) in larvae infected with SfNIC-B and SfNIC-Bpif1 rescue viruses and SfNIC-Begt and SfNIC-Bp10 recombinant viruses.

Hours post infection	Viruses			
	SfNIC-B	SfNIC-Bpif1	SfNIC-Begt	SfNIC-Bp10
0	-	-	-	-
2	-	-	-	-
4	-	-	130±10	-
6	-	-	734±74	-
8	-	-	1153±80	-
10	-	-	1939±101	-
12	104±9	101±6	5734±272	-
24	429±33	426±13	40241±4913	2584±154
48	1655±124	1439±69	180465±10284	180465±10284
72	2838±183	2559±176	351368±109814	1252025±109814

Transcript amplifications were performed using qSfBpif.F and qSfBpif1.R primers. Target gene copy numbers were calculated based on SfMNPV genome molecular mass and the standard curve. Values indicate means ± SD of three different repetitions measured twice for each sample.

qRT-PCR analysis of *pif1* was performed on total RNA extracted from larvae infected with the different viruses at indicated times post-infection.

Overall, *pif1* transcription was 123 to 137-fold higher or 441 to 489-fold higher in larvae infected with SfNIC-Begt or SfNIC-Bp10 at 72 h p.i. than in larvae infected with SfNIC-B or the rescue virus, respectively. Reprogramming of *pif1* transcription resulted in earlier and higher transcription for SfNIC-Begt and in later and extremely high transcription for the SfNIC-p10 virus.

### BV production occurred earlier in reprogrammed viruses

**Figure 2:**
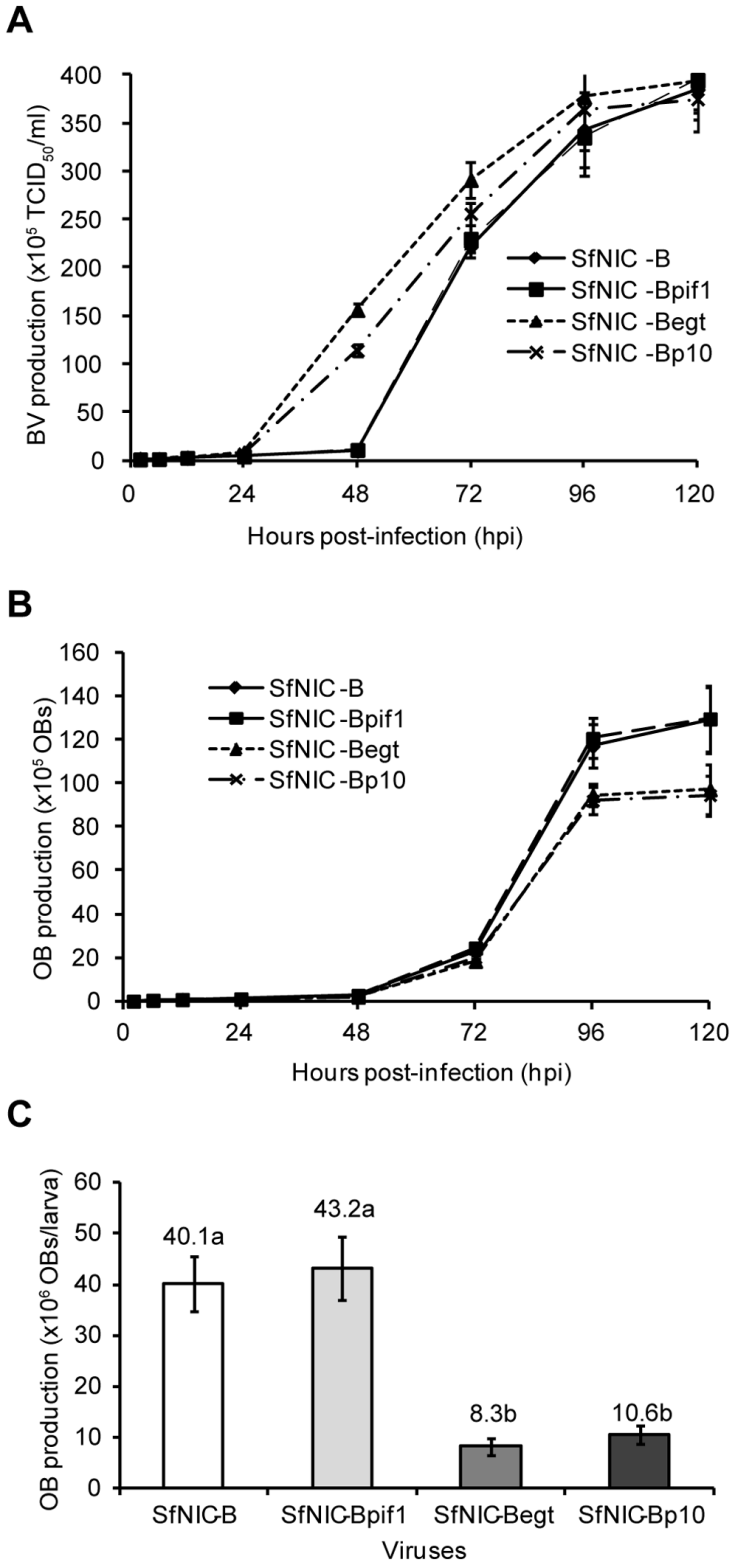
Virus production dynamics. A) Budded virus (BV) production in Sf9 cells by one-step growth curve analysis. 3×10^5^ cells were infected at 10 MOI using BV originating from SfNIC-B and SfNIC-Bpif1, SfNIC-Begt and SfNIC-Bp10 recombinant viruses. Supernatants harvested at indicated time points post infection were subjected to titer determination on Sf9 cells by end-point dilution. Each data point represents the average titer derived from three independent infections. The experiment was performed three times. Asterisks below hours-post infection values indicate significant differences in BV production between the viruses at indicated times. B) OB production values of SfNIC-B and SfNIC-Bpif1, SfNIC-Begt and SfNIC-Bp10 recombinant viruses at different times post infection in Sf9 cells (OBs/cell). Groups of 3×10^5^ cells were infected at 10 MOI with BV originating from SfNIC-B and SfNIC-Bpif1, SfNIC-Begt and SfNIC-Bp10 recombinant viruses. Cells were collected at indicated time points post infection and subjected to OB production determination by direct counting in a Neubauer hemocytometer. Each data point represents the average titer derived from three independent infections. C) OB production values of SfNIC-B and SfNIC-Bpif1, SfNIC-Begt and SfNIC-Bp10 recombinant viruses in *Spodoptera frugiperda* second instars (OBs/larva). Figures above columns indicate the value of each column. Values followed by different letters were differed significantly (ANOVA, Bonferroni test, P<0.05).

Virus growth curves from three independent experiments were compared for SfNIC-B and reprogrammed viruses (Fig. 2A). No significant differences were observed in the final BV titers among the different viruses. However, the growth curve kinetics differed significantly between viruses; BV production occurred earlier in cells infected with SfNIC-Begt or SfNIC-Bp10 compared to the parental or rescue viruses. No significant differences were observed in the BV titer of viruses at 2 h p.i. (Kruskal-Wallis K = 4.96, df = 3, p = 0.175), 6 h p.i. (F_3,32_ = 0.105, p = 0.957), or 12 h p.i. (F_3,32_ = 0.324, p = 0.808). By 24 h p.i. SfNIC-Bp10 and SfNIC-Begt presented significantly increased BV production (F_3,32_ = 14.1, p<0.001), whereas by 48 h p.i SfNIC-Bp10 and SfNIC-Begt produced approximately 11 and 15-fold more BVs, respectively, than SfNIC-B (F_3,32_ = 270, p<0.001). By 72 h p.i. BV titers were slightly higher in SfNIC-Begt and SfNIC-Bp10 infected cells (F_3,32_ = 4.89, p = 0.007), but at 96 h p.i. (F_3,32_ = 0.268, p = 0.853) and 120 h p.i. (F_3,32_ = 1.279, p = 0.298), BV production was similar among all viruses.

### Reprogramming *pif1* expression resulted in reduced OB potency and OB production but increased virulence

The biological activity of OBs was compared by lethal concentration metrics (LC_50_) and mean time to death analysis in *S. frugiperda* second instars. No significant differences were observed in the potency of SfNIC-B and SfNIC-Bpif1 rescue OBs (Table 2). Reprogramming the *pif1* promoter resulted in significantly lower potencies of SfNIC-Begt and SfNIC-Bp10 OBs, that were reduced by approximately 5 and 10-fold, respectively, compared to SfNIC-B or SfNIC-Bpif1 OBs. The differences in the relative potencies of SfNIC-Begt and SfNIC-Bp10 OBs were not statistically significant (Table 2).

**Table 2:** Probit regression and time to death analysis for SfNIC-Bpif1, SfNIC-Begt and SfNIC-Bp10 recombinant viruses compared with the SfNIC-B parental virus in *Spodoptera frugiperda* second instars.

Viruses	Intercept ± S.E.	LC_50_ (OBs/ml)	Relative potency	Fiducial limits (95%)		Mean time to death (h)	Fiducial limits (95%)	
				Low High			Low High	
SfNIC-B	−4.592±0.427	7.25×10^4^	1.00	-	-	124a	121	127
SfNIC-Bpif1	−5.296±0.467	6.22×10^4^	1.17	0.71	1.93	121a	118	124
SfNIC-Begt	−5.056±0.509	3.60×10^5^	0.20	0.11	0.37	100b	98	103
SfNIC-Bp10	−5.435±0.584	7.33×10^5^	0.10	0.05	0.19	99b	97	101

Probit regressions were fitted using the PoloPlus program [25]. A test for non-parallelism was not significant (χ^2^ = 2.61, d.f. = 3, P = 0.456). Lines were fitted with a common slope of 0.927±0.099 (S.E). Relative potencies were calculated as the ratio of effective concentrations relative to the SfNIC-B virus. Mean time to death (MTD) values were estimated by Weibull survival analysis [26]. MTD values labelled with different letters were significantly different (P<0.05). The hazard function (α) was 5.0577.

The mean time to death of insects infected with the rescue virus SfNIC-Bpif1 did not differ significantly from that of insects infected with SfNIC-B (Table 2), whereas insects infected by SfNIC-Begt and SfNIC-Bp10 died, on average, 21 - 25 h earlier than those infected by the wild-type or rescue viruses.

OB production was determined in Sf9 cells at intervals up to 120 h p.i. (Fig. 2B). No differences were observed in OB yields among the four viruses in the first 72 h p.i. However, at 120 h p.i. the cells infected by recombinant viruses with *pif1* expression driven by heterologous promoters produced approximately 25% fewer OBs than SfNIC-B or the SfNIC-Bpif1 rescue virus (F_3,32_ = 8.41, p<0.001).

OB production was also determined in *S. frugiperda* larvae (Fig. 2C). Total OB production/larva was approximately 4-fold lower in insects infected by SfNIC-Begt (8.20×10^6^ OBs/larva) and SfNIC-Bp10 (1.06×10^7^ OBs/ml), compared to those infected by SfNIC-B (4.01×10^7^ OBs/ml), or the rescue virus (4.32×10^7^ OBs/ml) (F_3,301_ = 23.002; p<0.001).

### Reprogramming *pif1* expression did not alter the physical characteristics of Obs

**Figure 3:**
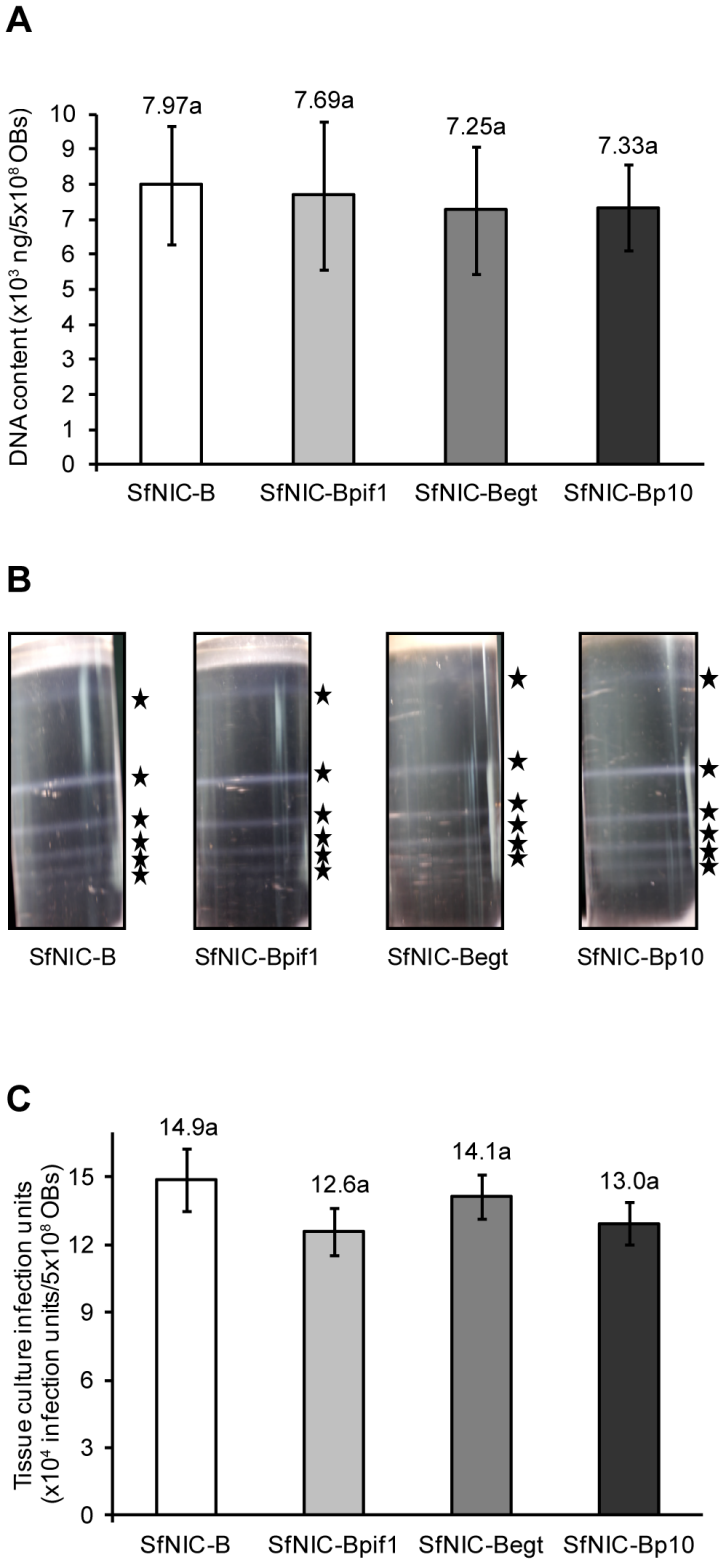
DNA and genome content of Occlusion Bodies. A) Mean amounts of DNA (ng/µl) extracted from samples of 5×10^8^ OBs of SfNIC-B, SfNIC-Bpif1, SfNIC-Begt and SfNIC-Bp10 viruses and determined by qPCR. Mean amounts of DNA from OB samples did not differ significantly between viruses (ANOVA, P = 0.401). B) ODV banding patterns of SfNIC-B, SfNIC-Bpif1, SfNIC-Begt and SfNIC-Bp10 viruses after sucrose-gradient separation of ODVs from similar quantities of occlusion bodies (5×10^8^ OBs). Stars indicate the positions of the observed ODV bands. C) ODV content in 5×10^8^ OBs of SfNIC-B, SfNIC-Bpif1, SfNIC-Begt and SfNIC-Bp10 viruses. Sf9 cells were infected with serial dilutions (1∶10, 1∶50, 1∶250, 1∶1250, 1∶6250) of ODVs released from OBs. ODV titers (ODV/ml) were calculated by end-point dilution. No significant differences were observed in the ODV content of OBs of the different viruses (ANOVA, P = 0.440).

Selected characteristics of OBs were determined in order to exclude them as a potential explanation for the observed differences in biological potencies of OBs among the *pif1* reprogrammed and parental viruses. No significant differences were detected by qPCR in the mean amounts of DNA in samples of 5×10^8^ OBs (F_3,104_ = 0.989, p = 0.401), suggesting similar numbers of genome copies in OBs of each of the different viruses (Fig. 3A). The efficiency of the qPCR technique was 99% (r^2^ = 0.9914). No gross differences were observed in the number of ODV bands or their intensity in samples originating from equal numbers of OBs of each of the viruses (Fig. 3B), indicating that these viruses did not differ appreciably in the distribution of numbers of nucleocapsids among ODVs. Finally, ODV titers from samples of 5×10^8^ OBs were estimated by end-point dilution (Fig. 3C), and did not differ significantly between any of the viruses tested (F_3,44_ = 0.919, p = 0.440).

### Reprogramming *pif1* expression shifted the composition of cooperator-defector mixtures in favor of defectors

**Figure 4:**
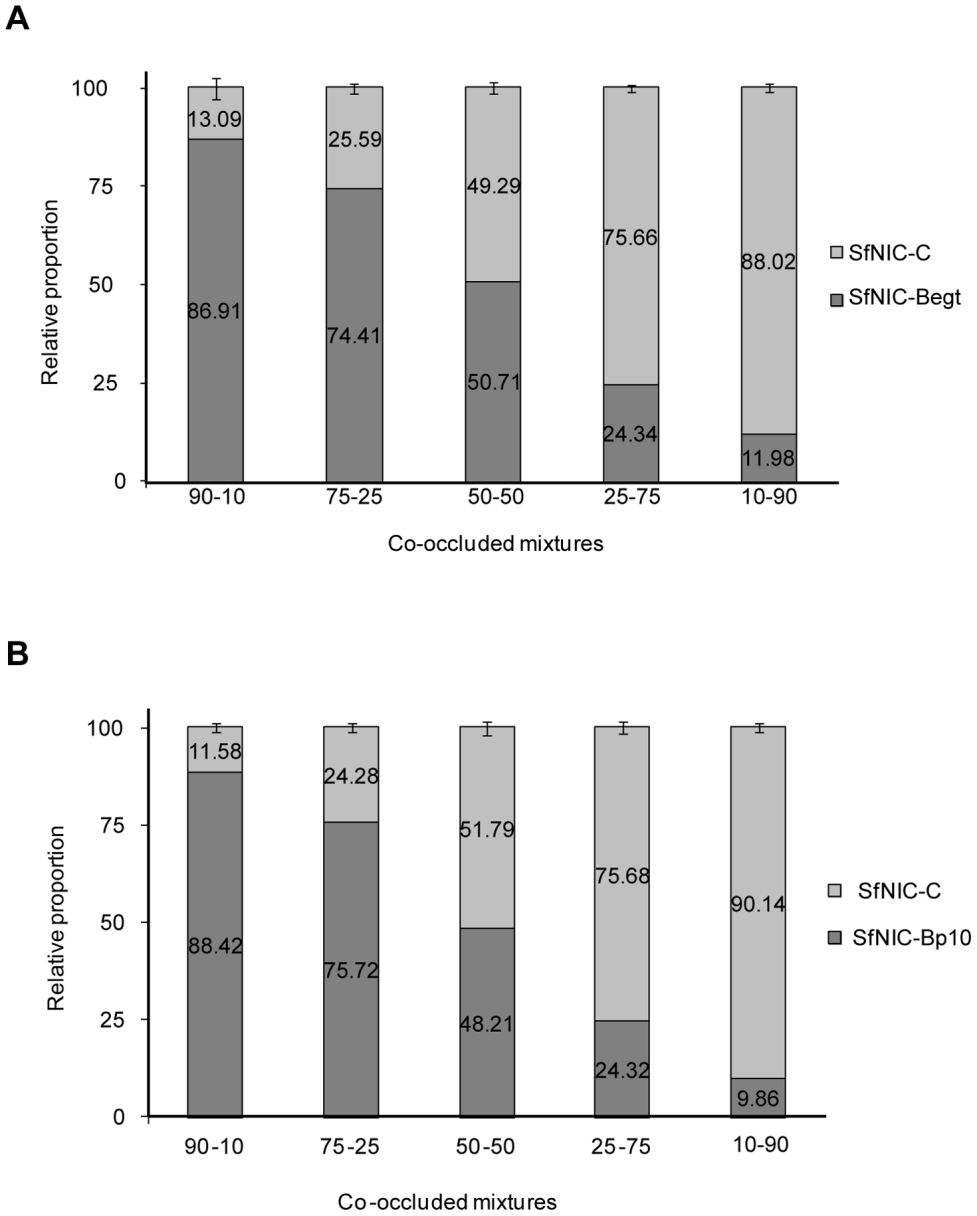
Relative proportions of SfNIC-Begt or SfNIC-Bp10 and SfNIC-C viruses in OBs comprising A) SfNIC-Begt:SfNIC-C and B) SfNIC-Bp10:SfNIC-C co-occluded mixtures at proportions of 90∶10, 75∶25, 50∶50, 25∶75 and 10∶90. The relative proportion of each virus was determined by qPCR using DNA extracted from co-occluded mixed genotype OBs obtained after injection of *S. frugiperda* fourth instars using ODVs released from OB mixtures at the desired proportions. Primers qSfBpif1.F and qSfBpif1.R were used for amplification of SfNIC-Begt and SfNIC-Bp10, that did not amplify in SfNIC-C. Primers qSfCcath1.F and qSfCsf361.R were used for amplification of SfNIC-C.

To determine whether reprogramming of *pif1* resulted in a shift in the proportions of genotype mixtures that resulted in a high potency OB phenotype, such as observed in the wild-type population, insects were injected with mixtures of ODVs from *pif1* reprogrammed virus and a natural defector genotype (SfNIC-C) in different proportions. The resulting co-occluded mixed genotype OBs comprising SfNIC-Begt:SfNIC-C and SfNIC-Bp10:SfNIC-C were analyzed by qPCR to confirm that genotypes were present at the proportions in which they were inoculated (Fig. 4). For qSfBpif1.F and qSfBpif1.R primers, qPCR efficiency was 98.0% (r^2^ = 0.9818), whereas for qSfCcath.F and qSfCsf36.F the efficiency was 97.7% (r^2^ = 0.9841). As observed previously, SfNIC-WT OBs were 2.65-fold more pathogenic than SfNIC-B OBs (Table 3). The potencies of OBs produced in larvae co-infected with mixtures containing 90, 25 and 10% of SfNIC-Begt were not significantly different from that of SfNIC-B alone. However the potency of mixed genotype OBs comprising 50% SfNIC-Begt +50% SfNIC-C (potency of 3.38) was equivalent to that of SfNIC-WT OBs (Table 3). Similar results were obtained with the mixtures involving the SfNIC-Bp10 virus; the potencies of OBs involving 90, 75, 25 and 10% of SfNIC-Bp10 did not differ significantly from that of SfNIC-B, whereas the co-occluded OB mixture comprising 50% of SfNIC-Bp10 +50% SfNIC-C (potency 2.99), was as potent as SfNIC-WT OBs (Table 3).

**Table 3:** Probit regression analysis of virus induced mortality in *Spodoptera frugiperda* second instars inoculated with (I.) Wild-type SfMNPV (SfNIC-WT) and purified variant SfNIC-B occlusion bodies (OBs). (II.) OBs comprising co-occluded mixtures of SfNIC-Begt and SfNIC-C defector genotype in the proportions indicated. (III.) OBs comprising co-occluded mixtures of SfNIC-Bp10 and SfNIC-C defector genotype in the proportions indicated.

Viruses	Intercept ± S.E.	LC_50_ (OBs/ml)	Relative potency	Fiducial limits (95%)	
				Low High	
(I.) SfNIC-B	−4.696±0.447	1.23×10^5^	1	-	-
SfNIC-WT	−4.719±0.431	4.65×10^4^	2.66	1.56	4.51
(II.) SfNIC-Begt + SfNIC-C					
90∶10	5.074±0.463	9.22×10^4^	1.34	0.79	2.29
75∶25∶00	−5.381±0.474	7.15×10^4^	1.73	1.03	2.89
50∶50	−5.339±0.467	3.65×10^4^	3.38	2.03	5.63
25∶75	−5.058±0.457	8.71×10^4^	1.42	0.83	2.41
10∶90	−4.934±0.463	1.48×10^5^	0.83	0.48	1.45
(III.) SfNIC-Bp10 + SfNIC-C					
90∶10	4.993±0.455	1.00×10^5^	1.23	0.72	2.11
75∶25	−5.104±0.457	8.28×10^4^	1.49	0.88	2.53
50∶50	−4.934±0.442	4.12×10^4^	2.99	1.78	5.04
25∶75	−5.192±0.467	1.05×10^5^	1.71	0.69	1.99
10∶90	−4.730±0.449	1.51×10^5^	0.82	0.46	1.44

Probit regressions were fitted using the PoloPlus program [25]. A test for non-parallelism was not significant (χ^2^ = 6.66, d.f. = 11, P = 0.826). Lines were fitted with a common slope of 1.022±0.093 (S.E). Relative potencies were calculated as the ratio of effective concentrations relative to the SfNIC-B virus that was assigned a nominal potency of 1.0.

## Discussion

In the present study, we hypothesized that the SfMNPV population was structured to optimize the prevalence of PIF1-producing genotypes (cooperators) in the infected cells and hence, in progeny OBs produced for virus transmission. Increasing the intracellular abundance of PIF1 due to higher expression by a cooperator genotype in infected cells would therefore favor an increased prevalence of the defector genotype. This was investigated by producing two recombinant viruses, each with *pif1* expression reprogrammed at its native locus using exogenous promoters. This approach has also proved useful for gene function analysis of other NPVs [29]–[31]. The *egt* and *p10* promoters were selected, as the *egt* gene is an early transcribed gene [16], [17], whereas *p10* is a very late and strongly transcribed gene [18], [19]. The transcription of *pif1* under its homologous promoter is extremely weak [8], as confirmed in the present study, which is likely to be responsible for the low quantity of PIF1 produced in infected cells [7]. The relative transcription level of *pif1* in insects infected with SfNIC-B or SfNIC-Bpif1 rescue viruses was ∼3.0×10^3^ cDNA copies/mg RNA at 72 h.p.i. When reprogrammed, *pif1* transcription was temporally-advanced (20h) and ∼130-fold higher with the SeMNPV *egt* promoter, whereas transcription was increased by ∼450-fold and delayed by 12 h when under the control of the SeMNPV *p10* promoter. These substantial modifications allowed us to examine the consequences on the potency of mixed genotype OBs at the population level.

The dynamics of BV production in *pif1*-reprogrammed viruses were temporally advanced compared to those of parental and rescue viruses, although final BV titers were similar among all viruses. Temporal shifts in the patterns of replication of *pif1*-modified virus were previously observed using a reporter gene based assay, which also indicated that final titers of PIF1 appeared to be similar in *pif1*-reprogrammed and parental viruses [32]. The reason for this is not clear. Modifying *pif1* expression might affect the temporal expression of other genes that are directly or indirectly related to BV production, such as observed in another NPV gene [33], or may modify the course of the infection.

The *pif1* reprogrammed viruses killed their hosts markedly faster than the SfNIC-B and rescue viruses, possibly due to the earlier onset of systemic infection in insects infected by the *pif1* reprogrammed viruses. BV production following ingestion of high doses of OBs determines the rate of spread of infection that is positively correlated with speed of kill in other baculoviruses [33]–[35]. As a result of the rapid demise of *pif1* reprogrammed virus-infected hosts, OB production in *pif1* reprogrammed viruses was reduced by one quarter *in vitro* and by ∼4-fold in insects compared to the parental and rescue viruses, reflecting the well-established tradeoff between speed of kill and OB production in baculovirus infected insects [36]–[39]. BV production was advanced by 48 hours in these reprogrammed viruses.

Improvement of the speed of kill has been one of the major research objectives for the development of recombinant baculoviruses as the basis for bioinsecticidal products. Two main approaches have been employed: the expression of insecticidal toxins, enzymes or hormones [40]–[43], the deletion of life-stage manipulating virus genes [44], or a combination of both [45]. In the present study, we demonstrated that a different approach based on the modification of the expression of a virus core gene resulted in a significant improvement in speed of kill. Although in the case of *pif1*, reprogramming of expression resulted in reduced OB potency that is undesirable for the development of virus insecticides, the concept of reprogramming viral gene expression opens diverse possibilities in the improvement of baculoviruses for pest control.

The potency of OBs produced by *pif1* reprogrammed viruses was approximately one logarithm lower than that of SfNIC-B and rescue viruses, in terms of concentration-mortality metrics. There could be two possible causes for this reduction in the insecticidal properties of OBs: first, that the physical composition of OBs was altered in reprogrammed viruses, although no significant differences were observed in the DNA content of OBs, distribution of numbers of nucleocapsids in ODVs, or the infectivity of the ODVs in cell culture. This leads us to favor the second hypothesis, that increased *pif1* expression reduced the infectivity of ODVs compared to parental and rescue viruses.

A marked increase in the abundance of *pif1* transcripts might result in an increase in the intracellular pool of PIF1. The resulting accumulation of PIF1 in ODVs may have influenced the functionality or integrity of the complex of PIF factors required for ODV infectivity during primary infection [5]. We suggest that this is likely to be the reason for the reduced potency of reprogrammed virus OBs in *per os* infected larvae.

Finally, in line with the concept that PIF1 concentration in ODVs is decisive in determining OB potency, co-occlusion of *pif1* reprogrammed virus and a *pif1/pif2* deficient genotype (SfNIC-C) resulted in an OB potency phenotype similar to that of the wild-type isolate, at a ratio of 50∶50 (cooperator: defector). As we predicted *a priori*, this ratio was shifted in favor of the defector genotype when compared to the 75∶25 mixture that previously restored wild-type potency to OBs comprising natural cooperator + defector genotypes (SfNIC-B + SfNIC-C, respectively) [12], [15]. In addition, as observed previously [13], [14], during five serial passages in larvae the proportions of each genotype in mixtures converged to an equilibrium ratio that maximized the likelihood of transmission. Moreover, once equilibrium frequencies of genotypes have been achieved, the proportions of genotypes in mixed genotype nucleopolyhedrovirus populations remains stable over successive passages [13], [14].

A significant proportion of ODVs contain a mixture of genotypes [24], and following ingestion of OBs multiple foci of primary infection are usually observed in the insect midgut [33]. These two factors favor transmission of a mixture of cooperator and defector genotypes. During the systemic phase of disease, each cell of a caterpillar is infected by multiple genomes (average 4.3 budded virions per cell) [46]. Such small group sizes tend to favor cooperative behavior among their members, as the costs of hosting defectors is proportionally higher than for large groups [47]. As PIF1 produced by cooperator genotypes was available to all genotypes in a particular cell, *pif1* expression appears to modulate group-specific fitness and therefore represents a cooperative trait.

Game theory models often predict maximal group fitness when defectors are absent [48], [49]. Some exceptions to this general rule include excess production of goods leading to inefficient use and diminishing benefits to group members [50]. In the case of our study, PIF1 production represents an unusual case in which group success (transmission) depends on production of this component, but overproduction is highly prejudicial to group-specific transmissibility, both in terms of OB pathogenicity and total OB yield from each infected insect. Essential goods are usually a source of competition, as each individual tries to maximize the amount they can acquire [51]. In our model, however, this required resource, PIF1, appears to be deleterious if present in amounts higher than required. As a result, the presence of defectors that effectively dilute the intercelluar pool of PIF1 is necessary and beneficial to the entire virus population. When *pif1* expression is manipulated, the level of defectors in the population shifts to compensate the variation in the amounts of PIF1 available. This is reflected in the genotypic composition of the OBs produced [13]. These results offer a new and unexpected perspective on cooperative behavior between viral genomes in response to the abundance of an essential public resource that is detrimental in excess.

## Supporting Information

Table S1 **Primers used in this study.**
(DOCX)
