# Supplementary material for: Expression of a Peroral Infection Factor Determines Pathogenicity and Population Structure in an Insect Virus
Source: PLoS One. 2013 Nov 5;8(11):e78834. doi: 10.1371/journal.pone.0078834 (PMC3818493; doi:10.1371/journal.pone.0078834)
Supplement: Table S1 — Primers used in this study. (DOCX) [file pone.0078834.s001.docx]

Table S1. Primers used in this study

| Primer | Sequence | Amplification purpose and location |
| --- | --- | --- |
| Sfarif1.1 | 5’-ATGTCAggTA  CcTTATCGGCATCCACTTGCAA-3’ | Left flanking region amplification. Forward primer located 1006 nt upstream the *pif2* ATG star codon (nt 31,228-31,247 in SfNIC-B genome). *Kpn*I restriction site is underlined. |
| Sfpif2.4 | 5’-atctgaggatCC  **TTTATAGACTCTTAGAG**AGATCTCACCGTCGGTATCGTGTTCACATCTCTCGG-3’ | Left flanking region amplification. Reverse primer located 1 nt upstream the *pif1* ATG start codon (nt 32,204-32,233 in SfNIC-B genome). A *Bgl*II and *Bam*HI restriction sites are underlined and the *pif1* promoter is shown in bold. |
| Sfpif1.12 | 5’- atctgaggatcc  AGAAAACATGGACAATGTCA-3’ | Right flanking region amplification. Forward primer located 1 nt downstream the *pif1* TGA stop codon (nt 35,038-35,057 in SfNIC-B genome). *Bgl*II restriction site is underlined. |
| Sffgf.1 | 5’- cgatcTAAGCTTA  TAAACGAGTGCGGATATGT-3’ | Right flanking region amplification. Reverse primer located 1038 nt downstream the *pif1* TGA stop codon (nt 36,056-36,075 in SfNIC-B genome). *Hind*III restriction site is underlined. |
| Sfpif2.5 | 5’-cgattgagatcT**A**  **TG**GTCACGATCGAGCGCGC-3’ | *pif2* gene amplification. Forward primer that amplified in *pif2* start codon (nt 32,234-32,253 in SfNIC-B genome). *Bam*HI restriction site is underlined. The ATG start codon is in bold. |
| Sfpif2.6 | 5’-tgaacTagatc**TT**  **a**GACGGGCGGCGAAGCTC-3’ | *pif2* gene amplification. Reverse primer located in the *pif2* stop codon (nt 33,411-33,430 in SfNIC-B genome). *Bam*HI restriction site is underlined. The TAA stop codon is in bold. |
| Sfpif1.13 | 5’-GTACACggatc  **ATG**TATAATATATTGTTGAT-3’ | *pif1* gene amplification. Forward primer that amplified in *pif1* start codon (nt 33,448-33,407 in SfNIC-B genome). *Bgl*II restriction site is underlined. The ATG start codon is in bold. |
| Sfpif1.14 | 5’-GCTGAggatccT  **ca**AACCACCGATATGTGGT-3’ | *pif1* gene amplification. Reverse primer located in the *pif1* stop codon (nt 35,018-35,037 in SfNIC-B genome). *Bgl*II restriction site is underlined. The TGA stop codon is in bold. |
| P1-0 | 5’-GCATCGAGATCT  AGTGTTCTTCTTATTATATTG-3’ | SeMNPV *egt* promoter amplification nt 26,828-26,948 in SeMNPV genome). *Bam*HI restriction site is underlined. |
| P2-0 | 5’-GCATCGGGATCC  GGTGACCGATGATTCG-3’ | SeMNPV *egt* promoter amplification (nt 26,933-26,948 in SeMNPV genome). *Bgl*II restriction site is underlined. |
| P10S | 5’-GATCT**ATAAG**TTT  ATTATTATAATTGTAATTATATTATACATTG-3’ | SeMNPV *p10* complementary forward promoter oligomer (nt 123,702-123,739 in SeMNPV genome). *Bgl*II restriction site after cutting is underlined. *p10* promoter is in bold. |
| P10AS | 5’-CATCCAATGTATA  ATATAATTACAATTATAATAATAAA**CTTAT**A-3’ | SeMNPV *p10* complementary reverse promoter oligomer (nt 123,702-123,739 in SeMNPV genome). *BamH*I restriction site after cutting is underlined. *p10* complementary promoter is in bold. |
| Sfpif1.7 | 5’-tcaccaccaacAC  acggacaac-3’ | Verification of the authenticity of the genomic modifications. Forward primer located 150 bp upstream the *pif1* start codon (nt 33,279-33298 in SfNIC-B genome). |
| Sfpif1.9 | 5’-cggttgacatcCT  atcggta-3’ | Verification of the authenticity of the genomic modifications. Reverse primer located 450 bp downstream the *pif1* start codon (nt 33,938-33,957 in SfNIC-B genome). |
| qSfBpif1.F | 5’-CTCACGCCGTGC  TCGACTCA-3’ | *pif1* transcription analysis (qRT-PCR) and quantification of the relative proportion of SfNIC-Begt and SfNIC-Bp10 in SfNIC-Begt:SfNIC-C and SfNIC-Bp10:SfNIC-C co-occluded mixtures. Forward primer that amplified 194 downstream *pif1* start codon (nt 33,640-33,659 in SfNIC-B genome). |
| qSfBpif1.R | 5’- CGTCGGTGATGG  TGATGATG-3’ | *pif1* transcription analysis (qRT-PCR) and quantification of the relative proportion (qPCR) of SfNIC-Begt and SfNIC-Bp10 in SfNIC-Begt:SfNIC-C and SfNIC-Bp10:SfNIC-C co-occluded mixtures. Reverse primer that amplified 272 downstream *pif1* start codon (nt 33,720-33,739 in SfNIC-B genome). |
| qSfCcath.F | 5’-TTATCTTGGCGCG  TCAACGC-3’ | Quantification of the relative proportion (qPCR) of SfNIC-C in SfNIC-Begt:SfNIC-C and SfNIC-Bp10:SfNIC-C co-occluded mixtures. Forward primer that amplified 51 nt upstream the deletion point in SfNIC-C genome (nt 18,701-18,720 in SfNIC-B genome). |
| qSfCsf36.R | 5’-AATCTTTTGCGTT  TAAGCAA-3’ | Quantification of the relative proportion (qPCR) of SfNIC-C in SfNIC-Begt:SfNIC-C and SfNIC-Bp10:SfNIC-C co-occluded mixtures. Forward primer that amplified 28 nt dowstrean the deletion point in SfNIC-C genome (nt 35,150-35,169 in SfNIC-B genome). |
